# Services for Adults With Autism Spectrum Disorder: a Systems Perspective

**DOI:** 10.1007/s11920-020-1136-7

**Published:** 2020-02-05

**Authors:** Paul T. Shattuck, Tamara Garfield, Anne M. Roux, Jessica E. Rast, Kristy Anderson, Elizabeth McGhee Hassrick, Alice Kuo

**Affiliations:** 1 A.J. Drexel Autism Institute, Drexel University, Philadelphia, PA USA; 2 UCLA School of Medicine, Los Angeles, CA USA

**Keywords:** Autism, Adulthood, Services

## Abstract

**Purpose of Review:**

We review original research about services for adults on the autism spectrum published from January 2013 through December 2018. The main aim is to characterize the topical and methodological aspects of research about services. We review research on services related to employment, living in the community, and social participation. We compare our results with those from a similar review published in 2012 to assess progress and identify where new directions in research about services for adults with autism are needed.

**Recent Findings:**

We found the evidence base about services for adults on the autism spectrum remains very small and highly variable in aims and methods. There is wide variability in methods used to define sampling frames and recruit participants. Most studies focus on employment. Almost no studies examine the overall ecosystem of services serving autistic adults. Few studies use a conceptual framework for understanding access to, or improvement of, services.

**Summary:**

The small size of the extant research coupled with inconsistent quality prevents the accumulation of new knowledge in ways that would significantly inform the improvement of systems of care for the growing population of adults on the autism spectrum.

## Introduction

Most of a typical life is spent in adulthood, yet only 2% of all autism research funding in the USA focuses on adult issues [1]. An ecological life course perspective highlights the need to improve systems of services and care as a key component of efforts to achieve better health and social outcomes for this population [2, 3••]. A sole research focus on developing individual-level treatments targeting things like social skills and behaviors while ignoring the social determinants of meaningful community participation will not move the needle on population-level outcomes. Evidence-based behavioral and skills interventions do not self-disseminate or self-organize into services and programs that are widely accessible across demographic segments and locales. Echoing the national research agenda on transition for youth with autism [3••], we recommend two overarching priorities for future research on services for adults: (1) identifying how community- and systems-level determinants influence outcomes that are observable at a population-level and (2) increasing meaningful involvement of a broad array of community stakeholders, including autistic advocates, in the improvement of service systems.

Autism spectrum disorder (ASD) is characterized by impaired social communication and interaction coupled with unusual or repetitive interests or behaviors to a degree that significantly impacts daily functioning [4]. The most recent Centers for Disease Control and Prevention estimate of the prevalence of autism among 8-year-old children in the USA is 16.8 per 1000 [5]. A recent survey of US households with children yielded an estimated prevalence of 25.0 per 1000 [6]. Multiplying prevalence by population estimates of the number of 17-year-olds in the USA, we estimate that between 70,700 and 111,600 youth on the autism spectrum will turn 18 years old this year—equating to roughly 707,000 to 1,116,000 over the next decade.

Autism prevalence estimates have steadily increased in recent decades with growing awareness and screening related to autism. Autism is conceptualized as a heterogeneous spectrum because people vary widely in terms of support needs, strengths, impairment levels in several domains, and the presence of co-occurring health and mental health issues. Many people on the autism spectrum will need special health care and supportive community services throughout adulthood. Services are embedded in institutional, financial, policy, and community contexts—an ecosystem of service provision.

This scoping review characterizes the topical and methodological aspects of research about services for adults (age 18 and older) on the autism spectrum published from 2013 through 2018. We focus on services related to employment, living in the community, and social participation. We compare our results with those from a similar review published in 2012 to assess progress and identify where new directions in research about services for adults with autism are needed [7••].

### Literature Review Methods

We sought studies about the provision of services to support outcomes related to employment, education, social engagement, and independence for adults with ASD. We limited the review to studies conducted in English-speaking countries (the USA, Canada, the UK, and Australia) and published between January 1, 2013 and December 31, 2018. We found articles by searching online databases and conducting forward and backward citation searches with relevant articles. Additional articles were nominated for consideration by experts.

We screened articles based on the following inclusion and exclusion criteria. The study had to be original research involving, or pertaining to, individuals on the autism spectrum over the age of 18. If a study also included participants under 18, then the reporting of results had to break out findings for those over 18 or explicitly state the primary focus was on adult services. We excluded reviews, research agenda recommendation studies, and workgroup proceedings. If a study included multiple disability populations, then there had to be findings broken out by type of disability so the autism-specific findings could be clearly discerned. We excluded articles that focused primarily on issues related to caregivers or family members of individuals with ASD. Many articles ambiguously used the term “transition-age youth” that included individuals 18 years of age or older. We excluded articles that focused mainly on services aimed at preparing those under age 18 for adult life. To be included, a primary purpose of a study on transition-age youth had to be describing or evaluating support systems, services or programs for young adults with ASD or evaluating the impact of services on outcomes related to employment, education, and inclusion in the community. We also included articles that focused on topics related to service delivery for adults with ASD such as service provider perspectives, financing of services, and needs assessments. Articles describing adult outcomes that did not include a primary focus on services were excluded. Studies that focused mainly on characterizing impairments or genetic, biological, physiological, and neurological characteristics associated with autism were also excluded from the review. We excluded clinical, behavioral, and medication interventions aimed at mental health, physical symptoms, cognitive functioning, or individual behaviors unless the study was focused on how to scale or implement the intervention at a population level.

This review focuses on research about services for adults age 18 or older. However, we note that it is difficult to define a clear boundary demarcating “adult” versus “transition” research literature. Increasing recognition of a period of “emerging adulthood” has shifted our understanding of the upper age boundary of the transition to adulthood period. Starting in the 1990s, a growing body of research found that the transition into adulthood takes longer in contemporary society and today’s youth oftentimes do not achieve independence until their late twenties [8]. The emerging adulthood stage of development is thought to occur between 18 and 29 years [9–11]. This reconceptualization is evident within recent federal and state regulations such as the Affordable Care Act that extended coverage to youth under their parents’ insurance until age 26. The Annie E Casey Foundation now reports statistics on employment for “young adults” ages 18–29. The Pew Research Center’s research on living arrangements of young adults investigates ages 18–34. This shift poses unique challenges in terms of producing a follow up review of adult services as we were faced with the challenge of discerning a clear boundary between the service architecture that is emerging to accommodate the transition period from what is historically referred to as “adult services.”

### Literature Review Findings

Fifty-two studies met the inclusion criteria (Table 1). To contextualize this number, we searched the PubMed database during the same time period using the keyword “autis*” and found 23,464 peer-reviewed studies. We experimented with other PubMed search parameters and consistently came up with roughly 23,000 published studies. Thus, the studies reviewed here represent less than 1% of scholarly output on autism from 2013 through 2018.

**Table 1:** Studies included in review

Study	Data source	Labels used to describe sampling frame and/or participants	Sample size	Age, years (mean)	Male %	Verbal %	White %	SEP
Employment								
Baldwin et al. [12]	Purposive sampling of participants from the We Belong Survey	Autism spectrum disorder, Asperger’s disorder, high functioning autism, PDD-NOS, autistic disorder	130	18–65	68	NR	NR	YES
Bross et al. [13]	Convenience sample recruited from a community college and nonprofit organization	Asperger syndrome	1	18	100	100	100	NR
Burgess et al. [14]	Nationwide RSA-911 data from 2002 to 2011	Autism spectrum disorder	34,501	(20.32) NR-22	82.2	NR	83.8	YES
Chen et al. [15]	Rehabilitation Service Administration Case Services Report (RSA-911) for the 2011 fiscal year.	Autism	5681	NR	84	NR	80.2	YES
Ditchman et al. [16]	RSA-911 data for 2009 fiscal year	Autism, autism spectrum disorder	2219	(18.55) 16–24	84.26	NR	82.2	NR
Gentry et al. [17]	Convenience sample from Virginia Department of Aging and Rehabilitative Services	Autism spectrum disorder	50	18–60	84	68	60	YES
Hill et al. [18]	Convenience sample of participants in the Triumph Project, an urban, nonprofit rehabilitation program	Autism spectrum disorders	3	23–26	33	NR	NR	NR
Kaya et al. [19]	RSA-911 data	Autism, autism spectrum disorder	4322	16–25	85	NR	79	YES
Kaya et al. [20]	RSA-911 data	Autism	3243	19–25	83	NR	78	YES
Migliore et al. [21]	RSA-911 data sampled for 19 states from 2006 to 2010	Autism	24,215	16–26	NR	NR	NR	NR
Morgan et al. [22]	Convenience sample, recruited through CARD system and local organizations	Autistic disorder, Asperger’s syndrome, pervasive developmental disorder—not otherwise specified, autism spectrum disorder	28	18–36	96	NC	86	YES
Nicholas et al. [23]	Convenience sample from autism programs and organizations then researchers used snowball sampling methods to recruit participants.	Asperger’s syndrome, pervasive developmental disorder not otherwise specified, and autism	71	18–65	69	NR	NR	NR
Schall et al. [24]	Retrospective record review of people who received supported employment services from a rehabilitation provider	Autism, autism disorder, Asperger’s disorder, PDD-NOS	45	(25.8)	82	NR	69	YES
Smith et al. [25]	Convenience sample	Non-specific autism spectrum diagnosis	26	(24.2) 18–31	76.9	NR	46.2	YES
Smith et al. [26]	Convenience sample	Non-specific autism spectrum diagnosis	23	(24.3)	73.9	NR	43.5	YES
Sung et al. [27]	RSA-911 data used: full sample of eligible women and random sample of 20% of men	Autism, autism disorder, Asperger’s disorder, PDD-NOS	1696	16–25	51	NR	80	YES
Wehman et al. [28]	Convenience sample	Autism, pervasive developmental disorder—not otherwise specified, or Asperger’s disorder	40	18–21.5	72.5	NC	46.7	NR
Wehman et al. [29]	Retrospective record review, convenience sample	Autism spectrum disorder	64	(26) 19–59	82	NR	63	YES
Postsecondary education								
Hendrickson et al. [30]	Program participants were sampled	Autism spectrum disorder	14	18–22	NR	NR	NR	NR
Koegel et al. [31]	Purposive instrumental sample	Autism spectrum disorder, Asperger’s syndrome	3	21–23	100	NR	66.7	NR
Rando et al. [32]	Convenience sample	Autism spectrum disorder	11	(19)	80	NR	NR	NR
Roux et al. [33]	NLTS2 data	Autism	620	21–25	85	10.9–63.5	85	YES
Siew et al. [34]	Convenience sample	Autistic disorder	10	17–20	70	NR	NR	NR
White et al. [35]	Convenience sample recruited through University office of disability services	Autism spectrum disorder	8	(20.5) 18–23	62.5	NR	87.5	NR
Employment/postsecondary								
Briel et al. [36]	Purposive sampling of students with ASD receiving Disability Support Services Offices at colleges within the state.	Autism spectrum disorder	18	18–43	83	NR	89	YES
Taylor et al. [37]	Sample from a larger longitudinal study	Autistic disorder, Asperger disorder, pervasive developmental disorder—not otherwise specified	161	18–52	72	30.4	NR	YES
Social skills								
Kandalaft et al. [38]	Convenience sample from another study on social cognition	Asperger syndrome or PDD-NOS	8	18–26	75	NR	87.5	NR
Laugeson et al. [39]	Convenience sample from UCLA PEERS clinic	Autism spectrum disorder	17	18–24	76.5	NR	52.9	NR
Mehling et al. [40]	Sampled from NCI survey	Autism spectrum disorder	886	(33.2)	73.9	NR	75.2	NR
Nuernberger et al. [41]	Convenience sample	Autism, Asperger’s syndrome	3	19–23	67	NR	NR	NR
Medical/Healthcare								
Liu et al. [42]	Data from healthcare claims	Autism spectrum disorder	87,683	12–21	79.7	NR	NR	NR
Nicolaidis et al. [43]	Online convenience sample	Autistic disorder, Asperger’s disorder, pervasive developmental disorder—not otherwise specified	209	18-NR	41	NR	86	YES
Nicolaidis et al. [44]	Purposive sampling for maximum variation	Autism, Asperger’s, pervasive developmental disorder not otherwise specified, or autism spectrum disorder	39	19–64	56	NR	64	YES
Vohra et al. [45]	Sample from records	Autistic disorder, Asperger’s syndrome, and other pervasive developmental disorders	25,257	22–64	75.5	NR	NR	YES
Weiss et al. [46]	Analysis of health administrative and disability income supports data from Ontario	Autism spectrum disorder	5095	18–24	77.6	NR	NR	NR
Organizational management/Staff or provider issues								
Warfield et al. [47]	Purposive sampling of physicians who provide medical care for adults with autism	NA	NA	NA	NA	NA	NA	NA
Zawacki et al. [48]	Convenience sample of staff members and adults receiving services	Autism spectrum disorder	3	20–27	0	NR	NR	NR
Zerbo et al. [49]	Convenience and purposive sampling	NA	NA	NA	NA	NA	NA	NA
Independent living skills								
Burckley et al. [50]	Convenience sample	Pervasive developmental disorder—not otherwise specified	1	18	0	NR	100	NR
Policy								
Williams [51]	Legal case study	Asperger’s disorder	1	NR	100	NR	NR	NR
Residential Services								
Hewitt et al. [52]	Disproportionate random sampling of 400 participants in 25 states with replacement	Autism spectrum disorder	1459	18-NR	77.4	NR	72.3	NR
Service Needs Assessment								
Anderson et al. [53]	Convenience sample	Autism spectrum disorder, autism, Asperger’s, PDD-NOS, other ASD	20	(24)	75	NR	90	YES
Farley et al. [54]	Sampled from a previous epidemiological survey	Autistic disorder, autism, autism spectrum disorder	162	22–51	76	NR	NR	NR
Koffer Miller et al. [55]	Convenience sample	Autism spectrum disorder	36	21-NR	69.9	NR	NR	NR
Lai et al. [56]	Recruited survey participants across Canada	Autism spectrum disorder, autism, Asperger syndrome, pervasive developmental disorder—not otherwise specified	3317	2–61	82	NR	84	YES
Mukherjee et al. [57]	Purposive sampling of service users from three service locations	Autism spectrum disorder	225	18–65+	68	NR	NR	NR
Sosnowy et al. [58]	Convenience sampling and snowball sampling	Autism spectrum disorder	20	18–29	41.2	NR	88.2	YES
Tint et al. [59]	Convenience sampling and snowball sampling	Autism spectrum disorder	20	19–69	0	NR	90	YES
Turcotte et al. [60]	Data from Pennsylvania Autism Needs Assessment survey	Autism spectrum disorder, Asperger’s disorder, autistic disorder, pervasive developmental disorder not otherwise specified	3269	(25)	81	NR	89	YES
Costs								
Buescher et al. [61]	Cost estimates calculated by compiling information from multiple studies	NA	NA	NA	NA	NA	NA	NA
Mavranezouli et al. [62]	Cost effectiveness analysis based on data from a prior study of supported employment intervention for adults on the 11111autism spectrum	NA	NA	NA	NA	NA	NA	NA
Social Participation								
Milton et al. [63]	Convenience sample	Autistic, autism spectrum	12	NR	NR	NR	NR	NR

Of the 52 studies, 42 took place in the USA, four in Canada, three in the UK, two in Australia, and one used data from both the USA and the UK. About one-fourth came from four teams. The Nicolaidis and Raymaker group did two studies related to healthcare. Smith et al. had two studies on virtual reality/employment interventions. Kaya et al. had two studies on employment and demographic/benefits related correlates. Wehman’s team authored five studies. Weiss collaborated with researchers on three studies related to service needs and healthcare for adults on the autism spectrum.

### Distribution of Topics and Aims

Over half (*n* = 27) the studies were about employment. Other focal topics included social participation (*n* = 15) and postsecondary education support programs for adults with autism (*n* = 11). Eight studies focused on healthcare services for adults on the autism spectrum. Seven studies included a focus on the cost of services. Five studies were related to behavioral services. Relatively few studies examined financial needs (*n* = 4) or independent living (*n* = 2). Two studies were about both employment and postsecondary education. One study focused on describing the characteristics of people with autism who use residential services.

Nine studies assessed the impact of interventions that included assistive technology (AT) [13, 17, 18, 25, 26, 29, 35, 38, 50]. Among the AT studies, six studied the use of AT to improve employment outcomes [13, 17, 18, 25, 26, 29], one involved the use of a computer-based intervention for college students [35], one involved using AT to support the development of social skills [38], and one involved using AT to teach community shopping skills [50].

Sixteen studies used large secondary datasets to examine questions at a population- or systems-level. Seven were based on data from the US Rehabilitation Services Administration system. Two studies included analysis of data from the National Core Indicators Adult Consumer Survey, and one study utilized data from the National Longitudinal Transition Study-2.

Nineteen studies involved the formal evaluation of an intervention, service, or program [13, 17, 18, 22, 24–26, 28–32, 34, 35, 38, 39, 41, 50, 63]. Nine were about employment [13, 17, 18, 22, 24–26, 28, 29], five about postsecondary support programs [30, 32, 34, 35, 63], and five about social participation [31, 38, 39, 41, 50].

### Diverse Approaches to Study Design, Wide Variability in Sample Characteristics

In studies using Rehabilitation Services Administration data, sample sizes ranged from 1696 to 34,501. The number of participants with ASD ranged from one to 87,683 in the remaining studies. Among the studies that did not use large administrative or national survey datasets, sample sizes ranged from one to 225 (mean 41).

Four studies did not involve data about participants on the autism spectrum—two examined physicians’ perspectives on providing healthcare services to adults with autism [47, 49] and two used secondary data to estimate costs or cost effectiveness of services [61•, 62]. Seven studies solely used qualitative methods [36, 44, 49, 53, 55, 58, 59] and two used mixed methods [23, 35].

Three of the 48 studies did not report the distribution of males and females [21, 30, 63]. The mean percentage of males was 78.5% in studies using large administrative or survey datasets and 65.8% in the remaining studies.

The majority (42 of 48) of studies that included participants with autism characterized the age of the ASD sample in some way. However, age information was incomplete in several instances. Three studies lacked specific details about the ages of ASD participants, other than noting participants were adults or that the services described were intended for adults [15, 51, 63]. One study used three age categories but the age range or mean age of participants was not reported [15]. In four studies, the upper age limit of the sample was not specified [43, 52, 55, 57].The lower boundary of the sample’s age range was not reported in one study [14]. In studies that reported the mean age of ASD samples, the means ranged from 19 to 33 [14, 16••, 24–26, 29, 32, 35, 40, 53, 60, 64].

Among 19 studies examining programs and interventions, two used single-subject designs [13, 50], seven used experimental designs [17, 22, 25, 26, 28, 35, 39], and ten used qualitative or non-experimental designs [18, 24, 29–32, 34, 38, 41, 63].

Forty-two studies provided information about how ASD status was determined. Eight studies specified that qualified professionals diagnosed participants [12, 37•, 38, 39, 48, 56•, 57, 59], and six studies utilized standardized measures to verify diagnoses [22, 25, 26, 35, 37•, 38]. Two studies included participants who did not have clinical diagnoses but self-identified as being on the spectrum [43, 44].

Severity of impairment was reported in ten articles using standardized measures of intellectual or adaptive functioning [13, 22, 28, 30, 35, 38, 39, 41, 50, 54]. Only four studies characterized the distribution of communication or verbal abilities in their samples [13, 17, 33, 37•]. Although most adults with autism have co-occurring health and mental health challenges, only 20 studies included information about the prevalence of co-occurring health and mental health conditions in their samples [12, 14, 15, 18, 27–29, 31, 35, 37•, 38, 40, 41, 43, 45, 46, 50, 52, 56•, 60].

### Reporting on Socioeconomic Position, Race, and Ethnicity

Socioeconomic position (SEP) describes the location of individuals and groups in a society’s social hierarchy based on characteristics including wealth, income, education, and occupation [65–67]. Twenty-five (52.1%) of the studies that included participants with autism did *not* characterize SEP for their sample [13, 16, 18, 21, 23, 28, 30–32, 34, 35, 38–42, 46, 48, 50–52, 54, 55, 57, 63]. The most frequently reported aspect of household SEP was autistic participants’ educational attainment (*n* = 16) [12, 14, 15, 17, 19, 20, 22, 24, 27, 29, 33, 36, 43••, 44, 58, 59]. Four studies included information about parental level of education [25, 26, 33, 58]. Only five studies reported household income [24, 33, 37, 53], and participants’ income was described in only one study [43••]. Three studies included information about participants’ receipt of public benefits (e.g., Medicaid, SSI, food stamps) [20, 27, 29]. One study included a measure of financial distress to assess the degree to which families were able to afford household expenses [56].

Thirty studies included a description of the racial composition of the sample. Eighteen studies included description of *both* racial and ethnic composition [14, 16•, 17, 19, 20, 22, 26, 29, 31, 33, 36, 43••, 44, 52, 53, 56, 58, 60] and, of those studies, only four [33, 43••, 52, 53] reported rate and ethnicity in a manner consistent with current federal guidelines [68]. None of the studies compared the racial-ethnic sample distribution to a population-based benchmark to characterize sampling bias.

### Conclusion and Considerations for Future Research

Similar to findings presented in a 2012 research review of adult services [7••], we found the evidence base about services for adults on the autism spectrum remains very small and highly variable in aims and methods. Most people with autism have co-occurring health, mental health, and social challenges that change as people age and require accessing services and supports from a wide range of providers. A life course systems perspective emphasizes the complex and evolving dynamics of interaction among culture, history, institutions, organizations, policies, funding, and families that impact individual development, service accessibility, delivery, coordination, and effectiveness [8, 69–85]. None of the reviewed studies adopted a systems perspective or produced new findings with immediate salience for improving complex systems of care and related outcomes. Systems thinking involves a focus of four core domains including the generation and dissemination of new information, network-based approach to facilitate collaboration within and across disciplines and organizations, the use of modeling strategies to guide decision-making processes strategically, and creating systemic change to promote better functioning and internal organization [86]. Systems perspectives have been adopted in public health practice and research as systems thinking facilitates greater understanding of how a complex range of components within health systems are structured, interacting, and functioning [86]. The systems perspective is essential to address complex public health challenges, such as improving services and outcomes for adults on the spectrum, which require coordinated interaction of multiple complex systems [86].

The wide variety of service-related topic areas identified in this review reflects the need for transdisciplinary systems research which could yield a more integrated understanding of how a diverse range of autism service systems function, coordinate, and can be strengthened or improved to promote better outcomes for adults on the autism spectrum. We found no studies rooted explicitly in improvement science—the study of identifying, implementing, evaluating, and disseminating strategies to bring about incremental improvements in system performance [87]. These approaches have a record of improving systems of care in other vulnerable populations and complex care ecosystems [74, 87–89, 90•, 91•, 92]. Most reviewed studies focused on a single intervention, program, or service system.

Future research needs to explicitly adopt frameworks for understanding, and interventions for improving, systems of care. Researchers have applied a systems perspective to understand how factors related to service systems, as well as individual and family-level factors, impact the transition to adult healthcare services for transition-age youth with disabilities. This systems-based approach was useful in identifying strategic systems changes which could promote better outcomes, and similar approaches could be used to improve autism service systems [93]. Future studies need to trace how individuals and families interact with multiple care team members simultaneously, whether care team members coordinate efforts and how to improve alignment of efforts and resource use across organizations.

Approaches to characterizing impairments and severity, sample demographics and socioeconomic position distributions, and co-occurring conditions were highly variable across studies and sometimes missing altogether. This emerging field would benefit from the development of consensus guidelines on study design and reporting standards. In the absence of reporting standards, it is very difficult to understand which subgroups and settings services-related research might generalize to.

The reviewed literature generally did not incorporate a contemporary life course perspective that considers the accumulation of disadvantage, or the impacts of opportunities, across the life span of autistic individuals. The life course health perspective frames health and functioning as interconnected and resulting from the complex interaction of multiple levels of determinants including biological, social, system, and economic contexts [82]. The life course consists of evolving social roles and shifts in the quality and availability of services that a person experiences over the course of their lifetime [82]. Future research could build on these findings by exploring disparities in pathways and trajectories across disability subgroups. Research that examines how gender, race, and social position intersect with disability is also needed. Prior research suggests that children from low-income households [94] and those with ASD [95] are separately at risk for poor outcomes, yet few studies have described the characteristics of children who meet both conditions. Although sociodemographic factors are generally considered to have low mutability [96], scholars have argued that the inclusion of such factors can help researchers identify and control for the effects of systems of social stratification [7••]. Specifically, sociodemographic variables can help to identify inequities in the distributions of services and resources across social groups, as well as help to identify the underlying mechanisms that give rise to such inequities [66].

AT was a focus in nine articles and was primarily used to develop employment-related skills. The emerging body of research evaluating the effectiveness of incorporating AT into services for adults on the autism spectrum suggests that technology can often facilitate the development of skills and promote increased independent functioning. That said, AT research with adults on the autism spectrum is still in an emergent state. States are now required to describe in their vocational rehabilitation state employment plans how a broad range of AT services and devices will be provided to improve vocational outcomes for people with disabilities. Further research is needed to identify the full range of potential applications of AT for adults on the spectrum and their association with positive outcomes for this population.

Healthcare for adults with ASD emerged in this review as a relatively new area of focus. Researchers utilized quantitative and qualitative data from a variety of sources, including physicians [47, 49] and adults with autism [43••, 44] to demonstrate that there is a significant need to improve physician training to serve this population. Further research is needed to determine how these findings can be translated into physician training and other efforts that will lead to better health outcomes for adults with ASD. Similar research approaches should be applied to better understand how adults with autism access and experience mental health services.

Most studies included information about sex, but description of gender and non-binary/transgender gender identities was not presented. Future work should be consistent with evolving understanding of gender and research on gender identity in adults with ASD [97]. As research related to gender identity in adults with ASD is emerging, researchers should use best practices for ascertaining gender identity [98] and develop strategies to recruit and include non-binary and transgender people with ASD in research.

Participants’ verbal ability was not characterized in the majority of reviewed studies. IQ was reported frequently but may have less of an impact on adult outcomes than other factors like communication ability and adaptive functioning, which were also often not reported. The persistent absence of a consistent and rigorous approach to measuring individual factors that influence outcomes inhibits the ability to determine which subgroups any given research findings may be relevant to.

The articles included in the review involved samples that were predominantly male and white. Greater efforts are needed to include racially and ethnically diverse samples in studies related to adults with ASD. Without this research, disparities cannot be identified or addressed.

The field of research on services for adults on the autism spectrum remains small and generally lacks unifying conceptual frameworks or consistent methodological approaches. These lacks undermine the potential for knowledge to accumulate and be applied to help specific subgroups of people. We recommend two overarching priorities for future research on services for adults: (1) identifying how community- and systems-level determinants influence outcomes and then measuring outcomes at a population-level and (2) increasing meaningful involvement of a broad array of community stakeholders, including autistic advocates, in the improvement of service systems. A study comparing employment-related service providers’ evaluations of their work to those of autistic adults and their families revealed that providers tended to have much more positive perceptions regarding the effectiveness of their services than people on the spectrum [23]. This study illustrates how different stakeholder groups can hold vastly different perspectives on the problems and relationships between components within a system. Focusing on a diverse range of stakeholder perspectives, including service providers, service users, and family members, in research on adult autism services could provide unique insight into system changes which could improve outcomes for this population. For example, a study included in the review that focused on healthcare experiences reveals that many autistic adults experience significant barriers to healthcare, highlighting a phenomenon that was previously not well-known in this population [44].

#### Funding information

This project was supported by the Health Resources and Services Administration (HRSA) of the US Department of Health and Human Services (HHS) under UJ2MC31073: Maternal and Child Health-Autism Transitions Research Project. This information or content and conclusions are those of the authors and should not be construed as the official position or policy of, nor should any endorsements be inferred by HRSA, HHS ,or the US Government.
